# In vitro eradication of citrullinated protein specific B-lymphocytes of rheumatoid arthritis patients by targeted bifunctional nanoparticles

**DOI:** 10.1186/s13075-016-0918-0

**Published:** 2016-01-16

**Authors:** Judit Pozsgay, Fruzsina Babos, Katalin Uray, Anna Magyar, Gergő Gyulai, Éva Kiss, György Nagy, Bernadette Rojkovich, Ferenc Hudecz, Gabriella Sármay

**Affiliations:** Department of Immunology, Eötvös Loránd University, Pázmány Péter sétány 1/c, Budapest, 1117 Hungary; MTA-ELTE Research Group of Peptide Chemistry, Hungarian Academy of Sciences, Eötvös Loránd University, Budapest, 1117 Hungary; Laboratory of Interfaces and Nanostructures, Institute of Chemistry, Eötvös Loránd University, Budapest, 1117 Hungary; Department of Rheumatology, Polyclinic of the Hospitaller Brothers of St. John of God, Budapest, 1023 Hungary; Department of Organic Chemistry, Eötvös Loránd University, Budapest, 1117 Hungary

**Keywords:** anti-citrullinated protein antibodies, B cell, citrullinated peptide, complement-dependent lysis, nanoparticles, rheumatoid arthritis, targeted therapy

## Abstract

**Background:**

Autoreactive B cells are crucial players in the pathogenesis of rheumatoid arthritis (RA). Autoantibodies specific for citrullinated proteins (ACPA), present in the serum of approximately 60–70 % of patients, have a pathogenic role in the disease. B cell depleting therapies may result in a transient immunosuppression, increasing the risk of infections. Our aim was to develop a new therapeutic approach to selectively deplete the ACPA producing autoreactive B cells.

**Methods:**

To target B cells synthetic citrullinated peptide derived from the β chain of fibrin, β60-74Cit _60,72,74_ (β60-74Cit), the predominant epitope recognized by ACPA was used. Complement dependent cytotoxicity (CDC) was induced by a modified peptide derived from gp120 of HIV-1. To trigger CDC both the targeting peptide and the complement activating peptide were covalently coupled in multiple copies to the surface of poly (DL-lactic-*co*-glycolic acid) nanoparticles (NPs). *Ex vivo* antibody synthesis was examined by ELISA and ELISpot. CDC was tested after dead cell staining by flow cytometry.

**Results:**

The β60-74Cit peptide was selectively recognized by a small subset of B cells from RA patients having high level of peptide specific serum antibody, suggesting that the peptide can target diseased B cells. The modified gp120 peptide covalently coupled to NPs induced the formation of the complement membrane attack complex, C5b-9 in human serum. We show here for the first time that bifunctional NPs coupled to multiple copies of both the targeting peptide and the complement activating effector peptide on their surface significantly reduce β60-74Cit peptide specific *ex vivo* ACPA production, by inducing complement dependent lysis of the citrullinated peptide specific B cells of seropositive RA patients.

**Conclusions:**

Bifunctional NPs covalently coupled to autoantigen epitope peptide and to a lytic peptide activating complement may specifically target and deplete the peptide specific autoreactive B-cells.

## Background

Rheumatoid arthritis (RA) is a chronic inflammatory autoimmune disease affecting mainly the small joints of the hands and feet causing severe tissue injury [1, 2]. RA has a worldwide distribution with an estimated prevalence of 0.5–0.75 %. Anti-citrullinated protein antibodies (ACPA) are present in approximately 60–70 % of RA patients. Citrullination is a posttranscriptional modification of arginine in certain proteins induced by peptidyl arginine-deiminase enzymes [3]. ACPA sometimes appear long before the manifestation of the disease [4], and therefore detection is not only the most specific and sensitive current diagnostic tool for RA [5] but also proved to have pathogenic significance. Several target proteins for ACPA have been described, such as citrullinated filaggrin [6–9], fibrin [10–13], vimentin [14–17], enolase [18–20], collagen [21–24], and certain viral proteins [25–27]. ACPA in the inflamed synovium have been shown to associate with citrullinated protein antigens such as fibrin to form immune complexes, leading to the progression of the inflammatory process [28, 29].

One of the most effective biological disease-modifying anti-rheumatic drugs is rituximab, a human CD20-specific chimeric monoclonal antibody, depleting CD20-bearing B cells [30–32]. After rituximab treatment, the B-cell number falls to an almost undetectable level at 3 months in most of the patients, and starts to rise at about 6 months [33]. Monitoring of serum antibody levels in rituximab-treated patients revealed that while titers of rheumatoid factor (RF) and anti-CCP antibodies significantly dropped, the humoral immune response towards most pathogens remained unaffected [34]. However, the transient absence of B cells may lead to an immunosuppressed state and leave the patients less protected against infections [35, 36].

Depletion of autoantigen-specific B-cell subsets recognizing the citrullinated protein epitopes might therefore be a more favorable therapeutic option. Citrulline (Cit)-containing peptides corresponding to ACPA epitopes may be recognized by circulating autoreactive B cells. We and others have shown that B cells from RA patients produce citrullinated epitope-specific antibodies ex vivo [37–40]. These data prompted us to introduce a Cit-containing peptide as a recognition unit to target specifically the autoreactive B cells.

The predominant synovial target for ACPA is the citrullinated fibrin [11–13]. Based on previous results identifying fibrin β60-74Cit_60,72,74_ (β60-74Cit) as the major epitope, we selected the corresponding synthetic peptide ^60^XPAPPPISGGGYXAX^74^ (X = Cit) for further experiments [10–12].

Complement-dependent lysis is one of the depletion mechanisms of B cells by rituximab, the most frequently applied B-cell depleting antibody [41, 42]. Therefore, we postulated that β60-74Cit peptide combined with a potentially complement activating peptide would induce specific lysis of the targeted epitope-specific B cells. Süsal et al. reported the complement activating capacity of a synthetic peptide derived from gp120 of human immunodeficiency virus type 1 (HIV-1) [43]. Based on this finding, we synthesized a novel peptide, Ac-^233^C(Acm)NNQTFNGTGPC(Acm)TNV^247^-K-NH_2_ (CNNQK), and then coupled it together with the targeting β60-74Cit peptide to the surface of carboxylate functionalized copolymer poly(d,l-lactic-*co*-glycolic acid) (PLGA) nanoparticles (NPs).

The results shown here revealed that these bifunctional NPs significantly reduced ACPA production in ex vivo culture by inducing complement-dependent lysis of citrullinated fibrin β-specific B cells.

## Methods

### Patients

For this cross-sectional study, blood samples were collected from 170 RA patients (146 women/24 men; median age 63 years, interquartile range 51–70; median disease duration 6 years, interquartile range 3–13) with established disease at the Buda Hospital, Budapest, Hungary of Hospitaller Brothers of St John. The diagnosis of the disease was established on the basis of the revised classification criteria of the American College of Rheumatology (ACR)/European League against Rheumatism (EULAR) [44].

Blood samples were taken with ethical permission and after the patients signed a written consent. The study has been approved by National Public Health and Medical Officer Service. Selected patients with a high level of β60-74Cit specific autoantibody (enzyme-linked immunosorbent assay (ELISA) ratios between 5 and 21 for β60-74Cit) were repeatedly recruited for the functional assays (Table 1). A total of 138 age-matched healthy control sera were obtained from the 3rd Department of Medicine, Semmelweis University, Budapest, Hungary.

**Table 1:** Clinical data of rheumatoid arthritis patients used in functional assays

	Gender	Age (years)	Disease duration (years)	CRP (mg/l)	RF (IU/ml)	ACPA (IU/ml)	DAS28	ESR (mm/h)	Treatment	β60-74 ELISA ratio
Patient 1	Female	54	5	8.1	264	1971	4.99	15	Sal, Afl, Lefl	10.26
Patient 2	Female	55	6	8.2	265	1971	4.1	16	Sal, Afl, Lefl	11.26
Patient 3	Female	78	9	1.84	16.2	2410	3.7	10	MTX	15.26
Patient 4	Female	82	12	2.1	10.9	Neg.	5.05	47	MTX, Med	0.89
Patient 5	Female	62	8	0.41	293.6	2403	2.17	21	Trexan	0.94
Patient 6	Male	45	6	0.7	47	3200	0.63	2	Del, Trexan	0.74
Patient 7	Female	52	4	3.1	264.1	3200	4.09	29	MTX, Med	6.59
Patient 8	Female	71	7	2.3	15	1759	2.83	19	MTX, Med	7.41
Patient 9	Female	39	6	0.5	96.2	3073	2.06	3	MTX	14.81
Patient 10	Female	45	3	1.9	394.5	295	1.1	3	MTX, Med	9.97
Patient 11	Female	43	3	2.5	10.3	952	1.05	6	Imuran, Metoject	7.65
Patient 12	Male	75	12	37.8	51.5	3200	2.5	4	Med, MTX	6.55
Patient 13	Male	74	19	0.14	360.9	2403	0.84	5	Roactemrat	13.8
Patient 14	Female	45	5	4.64	74.4	2991	4.01	9	Medrol, Trexan	20.97
Patient 15	Female	64	7	3.1	15.6	951	2.22	13	Lefl, Med	3.58
Patient 16	Female	63	5	6.7	673	964	2.1	20	Sal, Metoject	9.56
Patient 17	Male	70	9	9.3	25	3200	2.63	5	MTX, Sal, Med	14.35
Patient 18	Female	76	24	19.7	225	2132	7.97	42	Med, MTX	8.31
Patient 19	Female	41	1	2.3	129	765	3.41	4	Med	6.98
Patient 20	Female	60	9	47.86	65.3	349	5.39	97	Med, Nebivolol	6.35

*ACPA* anti-citrullinated protein antibodies, *Afl* aflamin, *CRP* C-reactive protein, *DAS28* Disease Activity Score of 28 joints, *Del* delagil, *ELISA* enzyme-linked immunosorbent assay, *ESR* erythrocyte sedimentation rate, *Lefl* leflunomid, *Med* medrol, *MTX* methotrexate, *Neg* negative, *RF* rheumatoid factor, *Sal* salazopyrin

### Peptide synthesis

All peptides were synthesized by solid-phase peptide synthesis as described previously [11, 38, 45]. The *N*-terminus of peptides was free or acetylated. *N*-terminally labeled β60-74 peptide amides (both β60-74Cit and β60-74Arg) were synthesized using biotinyl-aminohexanoic acid [38, 45]. The –COOH group on the *C*-terminus was always amidated (Table 2). Peptides were purified by semipreparative reversed-phase high-performance liquid chromatography (HPLC) and were characterized by analytical reversed-phase HPLC and electrospray ionization mass spectrometry (ESI-MS).

**Table 2:** Sequences of peptides used for the ELISA experiments and for the bifunctional nanoconstructs

Peptides	Code	Amino acid sequence^a^
Fibrinβ60–74	β60-74Arg	H-^60^RPAPPPISGGGYRAR^74^-NH_2_
Fibrinβ60–74Cit_60,72,74_	β60-74Cit	H-^60^ **X**PAPPPISGGGY**X**A**X** ^74^-NH_2_
Biot-fibrinβ60–74	Biot-β60-74Arg	Biot-^60^RPAPPPISGGGYRAR^74−^NH_2_
Biot-fibrinβ60–74Cit_60,72,74_	Biot-β60-74Cit	Biot-^60^ **X**PAPPPISGGGY**X**A**X** ^74^-NH_2_
HIV-1 gp120_233–247_ Complement activating peptide	CNNK	Ac-^233^C(Acm)NN**K**TFNGTGPC(Acm)TNV^247^-NH_2_
HIV-1 gp120_233–247_ Q-substituted complement activating peptide	CNNQ	Ac-^233^C(Acm)NN**Q**TFNGTGPC(Acm)TNV^247^-NH_2_
HIV-1 gp120_233–247_ Q-substituted complement activating peptide with C-terminal K	CNNQK	Ac-^233^C(Acm)NN**Q**TFNGTGPC(Acm)TNV^247^-K-NH_2_

^a^Standard one-letter code for amino acid residues: *X* citrulline, *Ac–* acetyl group, *–NH*_*2*_ amid group, *Acm* acetamidomethyl group, *Biot* biotinyl-aminohexanoyl group

*ELISA* enzyme-linked immunosorbent assay, *HIV-1* human immunodeficiency virus type 1

Bold letters stand for modified amino acids that are not present in the natural sequences

### Preparation and characterization of bifunctional PLGA NPs

Carboxylate-functionalized PLGA NPs were prepared by the nanoprecipitation method [46, 47]. The average hydrodynamic diameter, polydispersity, and zeta potential of PLGA NPs were characterized by dynamic light scattering and zeta potential measurements. The NPs were dispersed in doubly distilled water, finally containing 1.6 × 10^11^ NPs/ml. The average diameter of NPs was 160–180 nm, and each NP contained approximately 4–5000 carboxyl groups available for covalent binding of peptides.

NPs were converted in Milli-Q, Merck KGaA, Darmstadt, Germany water to PLGA-active ester derivative with high excess *N*-hydroxysuccinimide and 1-ethyl-3-(3-dimethylaminopropyl)-carbodiimide. The *N*-hydroxysuccinimide-activated particles were covalently linked to a 1:1 (mol:mol) mixture of CNNQK and β60-74Cit peptides. It should be noted that the targeting β60-74Cit peptide was coupled at its *N*-terminal α-amino group, while the CNNQK peptide was coupled at its *C*-terminal ε-amino group of Lys residue to the NPs. This attachment strategy resulted in uniformly oriented peptides of the two types. The efficient coupling of peptides to NPs was controlled after enzymatic digestion by HPLC/mass spectrometry (data not shown).

### B-cell purification

Peripheral blood mononuclear cells (PBMCs) were isolated from healthy donors and RA patients as described previously [38]. For the peptide-specific antibody secretion assay, B cells were purified by negative selection using RosetteSep antibody cocktail (Stem Cell Technologies, Vancouver, BC, Canada), according to the manufacturer’s instructions. The purity of the resulting B-cell population was 80–85 %. To stimulate antibody secretion, 10^6^ B cells/ml were cultured for 5 days in the presence of 7.5 μg/ml phosphorothioated unmethylated CpG oligodeoxynucleotide (ODN-2006, 5′-tcgtcgttttgtcgttttgtcgt′-3′; Sigma Aldrich Co., St. Louis, MO, USA) and 1.5 ng/ml human recombinant B-cell activating factor of the tumor necrosis family (BAFF; ImmunoTools GmbH, Friesoythe, Germany). The combined stimuli with CpG and BAFF were applied since Toll-like receptor TLR9 activation by CpG renders human B cells more sensitive to the effects of BAFF by increasing the membrane-bound BAFF that may enhance B-cell proliferation, differentiation, and autoantibody production [48].

A higher purity of B cells is needed for the peptide-binding and for the cytotoxicity assays. B cells were therefore purified by negative selection using magnetic bead-activated cell sorting (MACS) according to the manufacturer’s protocol (Miltenyi Biotec, Auburn, CA, USA). The purity of the resulting B-cell population was 85–98 %. B cells (10^6^/ml) were cultured with 1 μg/ml CpG, 50 ng/ml interleukin (IL)-2, and 50 ng/ml IL-21 (ImmunoTools GmbH) for 48 hours to increase the frequency of activated memory B cells.

### ELISA

In the serum ELISA, *N*-terminally biotinylated β60-74Cit and β60-74Arg were used. Biotinylated peptides (1 μg/ml in phosphate-buffered saline (PBS)) were bound to a NeutrAvidin (5 μg/ml in PBS; Pierce Biotechnology, Rockford, IL, USA) precoated plate [45]. ELISA ratios were calculated (optical density (OD) with β60-74Cit/OD with β60-74Arg) and compared between groups. The cutoff value was calculated from ELISA ratios of 138 healthy samples (means of ELISA ratios ± 2*standard deviation (SD)).

Antibody secretion was determined by ELISA from the supernatant of in vitro stimulated B cells, with plates coated as already described. The peptide-specific antibodies were detected by horseradish peroxidase (HRP)-conjugated anti-human IgG + M (Jackson Immunoresearch Laboratories Inc., West Grove, PA, USA).

### Detection of β60-74Cit peptide-specific B cells by flow cytometry

Purified B cells were cultured with 1 μg/ml CpG and 10 ng/ml IL-2 for 48 hours to increase the frequency of memory B cells. The biotinylated β60-74Cit or β60-74Arg peptides were coupled to NeutrAvidin-labeled yellow–green microspheres (1 μm diameter; Thermo Fisher Scientific Inc., Waltham, MA, USA). The peptide-coated fluorescent microspheres were added to B cells at 100-fold excess and the samples were incubated for 1 hour at 4 °C. The peptide-specific B cells were detected with a FACSCalibur flow cytometer (Becton-Dickinson, Franklin Lakes, NJ, USA) and data were analyzed using FlowJo software (Tree Star Inc., Ashland, OR, USA).

### Detection of IgG secreting cells by enzyme-linked immunospot assay

PBMCs were cultured in RPMI-1640 containing 10 % fetal calf serum in the presence of 10 ng/ml recombinant IL-2 and 1 μg/ml R848 polyclonal activator provided with the enzyme-linked immunospot assay (ELISpot) kit (Mabtech, Stockholm, Sweden). The cells were harvested at the 3rd day, and incubated for 1 hour with the bifunctional PLGA NPs at 1000-fold excess. After removing the unbound NPs, the cells were incubated at 37 °C for 30 minutes in the presence of 1 %, 3 %, 5 % and 10 % pooled normal human serum (NHS) as a complement source or in heat-inactivated serum. After washing, 4 × 10^5^ PBMCs were transferred into the wells of ELISpot plates precoated with the β60-74Cit peptides and with anti-IgG, respectively. The spots were developed after 18 hours by biotinylated detection monoclonal antibodies, streptavidin HRP, and substrate (Mabtech). The frequency of IgG-specific and peptide-specific IgG-producing cells was determined using a C.T.L. Immunospot analyzer (CTL-Europe GmbH, Bonn, Germany).

### Measuring the complement activating capacity of the modified HIV-1 gp120 peptides

Complement activation by the modified gp120 peptides ^233^C(Acm)NNKTFNGTGPC(Acm)TN^247^ (CNNK) and ^233^C(Acm)NNQTFNGTGPC(Acm)TNV^247^ (CNNQ) (Table 2) was tested by ELISA. High-binding ELISA plates (Greiner bio-one GmbH, Frickenhausen, Germany) were coated with 10 μg/ml peptide or with 100 μg/ml IgG as a control. Pooled human serum or heat-inactivated serum was added at 1:50 dilution for 1 hour. The deposited complement C3 cleavage products were detected by HRP-conjugated anti-human-C3 (MP Biomedicals, Solon, OH, USA), while C4 and C9 were detected by anti-human C4 and C9 antibodies (Merck KGaA, Darmstadt, Germany), respectively, followed by HRP-conjugated anti-goat IgG. The C1q depleted serum and the C3-depleted serum was purchased from Merck KGaA.

To test the complement activating capacity of the CNNQK-coated and β60-74Cit-coated bifunctional NPs, the release of the soluble complement components (SC5b-9) was measured. Then 10^9^ NPs were incubated in 100 μl 50 % human serum for 1 hour at 37 °C and SC5b-9 was determined in the supernatant by the SC5b-9 Plus kit (Quidel, San Diego, CA, USA) according to the manufacturer’s instruction.

### Cytotoxicity assay

B cells (10^6^ B cells/ml) were prestimulated with 1 μg/ml CpG and 10 ng/ml IL-2 for 48 hours. The bifunctional, fluorescein-containing NPs covered with the targeting β60-74Cit and the effector CNNQK peptides were added to B cells at 5000-fold excess, and then the samples were incubated for 1 hour on ice. After washing, the cells were incubated with 20 % pooled human serum as a complement source or heat-inactivated serum for 30 minutes at 37 °C, and then were stained with TO-PRO®-3 (Thermo Fisher Scientific Inc.) for 15 minutes to detect damaged cells. The dead β60-74Cit peptide-specific B cells were detected as the FL1/FL4 double-positive population with a FACSCalibur flow cytometer (Becton-Dickinson), and data were analyzed using FlowJo software (Tree Star Inc.).

### Statistical analysis

**Fig. 1:**
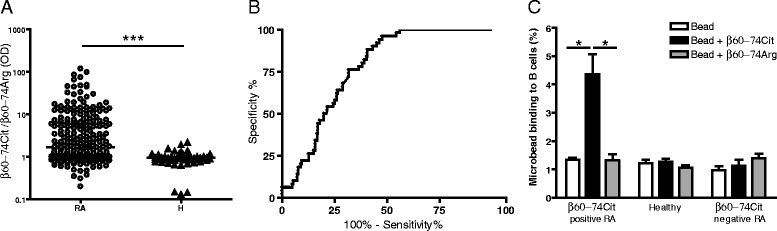
Recognition of Cit-containing peptide epitope of fibrin β chain by antibodies in sera of RA patients and healthy blood donors **a**, **b** and by isolated B cells **c**. **a** Reactivities of RA (*n* = 170) or healthy (*n* = 138) serum samples with N-terminally bitotinylated β60-74Cit vs. β60-74Arg bound to neutravidin precoated plates. ELISA ratios were calculated (OD with β60-74Cit /OD with β60-74Arg). Data were analyzed with the Mann–Whitney test, and the median OD ratio of RA samples was 1.68, interquartile range 0.95–6.33, and the median OD ratio of healthy samples was 0.91, interquartile range 0.81–1.07 (***p <0.001). **b** Receiver operating characteristic curve analysis, area under the curve value for β60-74Cit: 0.7661. **c** Binding of β60-74Cit and β60-74Arg-coated fluorescent microspheres to prestimulated B cells from β60-74Cit seropositive or seronegative RA patients and from healthy individuals (*n* = 3/group, ELISA ratios of the selected β60-74Cit seropositive RA patients were between 10 and 15). Bars show means ± SD, *p <0.05. *OD* optical density, *RA* rheumatoid arthritis, *H* healthy

**Fig. 2:**
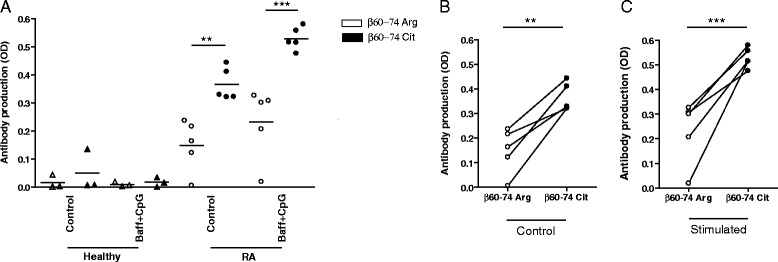
β60-74Cit peptide-specific antibody secretion of purified B cells from healthy donors and RA patients. B cells were cultured for 5 days in the presence or absence of 7.5 μg/ml CpG and 1.5 ng/ml BAFF **a** Antibody reactivities in the supernatants against β60-74Cit (*filled symbols*) and β60-74Arg (*open symbols*) peptides were detected by ELISA. Individual data points of unstimulated **b** and stimulated **c** samples are shown. The ELISA ratios of serum samples from the same donors were between 6 and 14. *n*_healthy_ = 3, *n*_RA_ = 5. The data were analyzed with the paired *t* test, means ± SD are shown (**p <0.01, ***p <0.001). *OD* optical density, *RA* rheumatoid arthritis

**Fig. 3:**
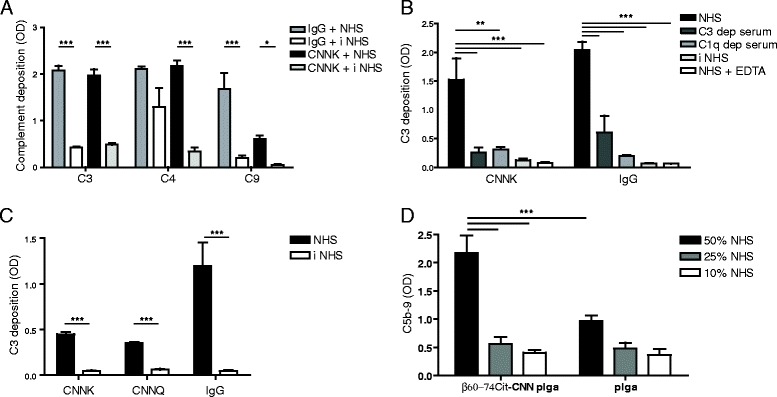
Complement activating capacities of HIV-1 gp120 derivative peptides CNNK and CNNQ and the NP-coupled CNNQK. **a** Pooled normal human sera (*NHS*) or inactivated sera were added to peptide-coated or IgG-coated plates. The deposited complement components C3, C4, and C9 were detected by anti-human C3-HRP, and anti-human C4 and C9 followed by HRP-conjugated anti-goat IgG, respectively. **b** C3 deposition from NHS is compared with that of C3 or C1q depleted serum and from NHS inactivated with heat or EDTA. **c** Comparison of C3 deposition induced by CNNK and the modified CNNQ peptide, and by human IgG. **d** CNNQK and β60-74Cit peptide-coupled NPs activate the C5b-9 terminal complex formation in human serum. Statistics: **a**, **b**, **d** analyzed with ANOVA, **c** analyzed with paired *t* test, means ± SD are shown (*p <0.05, **p <0.01, ***p <0.001). *EDTA* ethylenediamine tetraacetic acid, *OD* optical density, *PLGA* poly(d,l-lactic-*co*-glycolic acid), *RA* rheumatoid arthritis

**Fig. 4:**
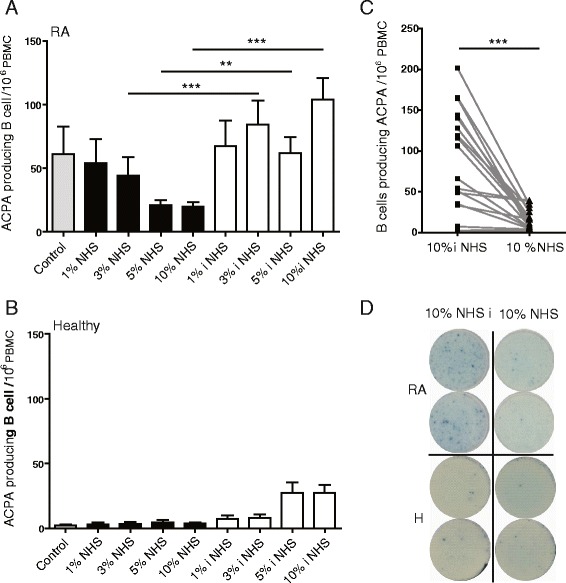
β60-74Cit and CNNQK peptide-coated bifunctional NPs suppress ex vivo synthesis of β60-74Cit specific antibodies in the presence of active complement in human sera. **a** PBMCs from RA patients (*n* = 17, ELISA ratios for β60-74Cit between 6 and 21) and **b** from healthy donors (*n* = 5) were stimulated with the TLR7/TLR8 agonist, R848, and IL-2 for 72 hours, and then the bifunctional NPs were added to the harvested cells followed by normal human sera (*NHS*) or heat-inactivated NHS (*iNHS*) at different concentrations. The number of β60-74Cit-specific IgG-producing plasma cells was counted by ELISPot. **c** Individual data of RA patients at 10 % iNHS and NHS are shown. **d** Representative ELISpot images. Statistics: **a**, c analyzed with paired *t* test, means ± SD are shown (**p <0.01, ***p <0.001). *ACPA* anti-citrullinated protein antibody, *PBMC* peripheral blood mononuclear cell, *RA* rheumatoid arthritis, *H* healthy

**Fig. 5:**
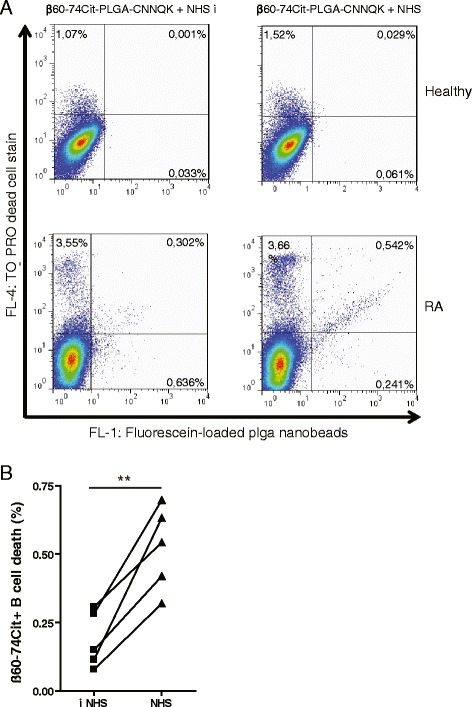
Complement-dependent lysis of β60-74Cit peptide-specific B cells induced by bifunctional PLGA NPs in the presence of NHS as measured by flow cytometry. **a** *Upper panel*: representative figure of a healthy donor, *lower panel*: typical result with an RA patient. The binding of fluorescein-loaded NPs was detected in channel 1, dead cells were detected after TO-PRO staining in channel 4. The β60-74Cit-positive, killed B cells are detected in the right upper quadrant of the dot plot. **b** Individual data of five RA patients. ELISA ratios of sera of the same RA patients were between 5 and 19. Data were analyzed with paired *t* test, means ± SD are shown (**p <0.01). *iNHS* heat-inactivated normal human sera, *NHS* normal human sera, *PLGA* poly(d,l-lactic-*co*-glycolic acid)

For the statistical analysis of data, the Mann–Whitney test (Fig. 1a), analysis of variance (ANOVA) (Figs. 1c, 2 and 3a, b, d) and the two-tailed *t* test (Figs. 3c, 4 and 5) were used and the results were analyzed with GRAPHPAD PRISM 4 software (GraphPad Software, La Jolla, CA, USA). In all tests, *p* <0.05 was considered significant.

## Results

### Recognition of Cit-containing fibrin β peptide by serum antibodies and by B cells of RA patients

Sera samples of 170 diagnosed RA patients and 138 healthy blood donors were screened by indirect ELISA. The ELISA ratios and the receiver operating characteristic curve are shown in Fig. 1a and b, respectively. With our experimental set up, at a 95 % specificity level β60-74Cit peptide was recognized by serum antibodies from 52 % of RA patients.

The presence of the serum antibodies specific for β60-74Cit suggests that we should find memory B cells in the blood of RA patients with identical specificity. To increase the binding avidity of the peptide we applied neutravidin-labeled polystyrene microspheres (1 μm diameter) loaded with a high-intensity fluorescent dye, coating its surface with Biot-β60-74Cit or Biot-β60-74Arg. B cells from selected anti-β60-74Cit peptide-positive or peptide-negative RA patients and from healthy controls were prestimulated to increase the frequency of memory B cells [49]. Microspheres coated with β60-74Arg bound to B cells at the same level as the uncoated controls, while microspheres covered with the β60-74Cit peptide specifically bound to a small but significant proportion of B cells from RA patients, but not to B cells from healthy volunteers, or from β60-74Cit antibody-negative patients (Fig. 1c).

### In vitro secretion of β60-74Cit peptide-specific antibodies by B cells of RA patients

To reveal whether ex vivo stimulation of B cells results in β60-74Cit peptide-specific antibody secretion, purified B cells from selected RA patients with high serum antibody level and from healthy controls were cultured for 5 days in the presence of the TLR9 agonist, CpG ODN, and BAFF. Antibody production was followed by testing the presence of native β60-74Arg-specific and β60-74Cit-specific IgG in the culture supernatants. B cells from healthy individuals did not produce a considerable amount of peptide-specific antibodies, while the patients’ B cells secreted IgG specific for the β60-74Cit peptide even without stimulation. A significantly lower level of β60-74Arg specific IgG was detected in all samples. CpG and BAFF double stimuli increased the β60-74Cit peptide-specific IgG secretion (Fig. 2a). Individual data sets are shown in Fig. 2b (unstimulated cells) and Fig. 2c (B cells stimulated with CpG and BAFF).

### Analysis of complement activating capacity of the modified gp120-derived synthetic peptides and the bifunctional NPs

In order to induce complement-dependent lysis of autoreactive B cells, our aim was to combine the targeting properties of β60-74Cit peptide with a peptide inducing complement activation. We shortened the corresponding gp120 peptide [43] at the C-terminal end with protected Cys residues and obtained the peptide CNNK (Table 2). CNNK had a comparable complement activating property with IgG regarding C3 and C4 deposition, while a somewhat lower level of C9 deposition was detected that was still significantly higher as compared with heat-inactivated sera (Fig. 3a).

C1q is essential to trigger the classical complement activation pathway. Neither the CNNK peptide nor IgG induced deposition of C3 from C1q-depleted as well as from C3-depleted sera, indicating that the classical pathway is activated. EDTA by chelating Ca^2+^ inhibited complement activation, and thereby C3 deposition (Fig. 3b).

For the coupling of CNNK peptide to the surface of NPs first we had to substitute 236Lys with Gln. The new peptide CNNQ had identical reactivity with CNNK, both inducing C3 deposition on peptide-coated surfaces (Fig. 3c).

CNNQ peptide was coupled to NPs via the ε amino group of an added C-terminal Lys (CNNQK). The complement activation property of the bifunctional NPs covalently coupled to both β60-74Cit and CNNQK peptides was tested by monitoring the formation of soluble C5b-9 terminal complex in human serum (Fig. 3d). Bifunctional NPs induced a significantly higher C5b-9 release as compared with noncoated NPs, indicating that the former activate the terminal components of complement system.

### β60-74Cit and CNNQK peptide-coated bifunctional NPs suppress β60-74Cit-specific antibody synthesis

To study whether bifunctional NPs indeed target B cells that specifically recognize the β60-74Cit peptide, PBMCs from selected RA patients having high β60-74Cit peptide-specific serum antibody level and from healthy donors were stimulated with the TLR7/TLR8 agonist, R848, and IL-2 for 72 hours, and then the harvested cells were incubated with the bifunctional NPs, followed by the addition of pooled human sera or inactivated sera at different concentrations. The number of peptide-specific IgG-producing plasma cells was estimated by ELISpot on the next day. Stimulation of B cells from RA patients but not from healthy individuals by TLR-mediated signals induced the differentiation of plasma cells that secreted β60-74Cit peptide-specific antibodies (Fig. 4a, b). In the presence of active complement, depending on serum concentration, the bifunctional NPs significantly reduced the number of β60-74Cit peptide-specific plasma cells, while in heat-inactivated serum NPs had no effect (Fig. 4a). The individual peptide-specific spot numbers in samples treated with 10 % heat-inactivated or active sera (Fig. 4c) and representative ELISpot images (Fig. 4d) are shown. Nonspecific IgG secretion was not affected by the bifunctional NPs (data not shown).

### The bifunctional NPs induce complement-dependent lysis of β60-74Cit peptide-specific B cells

Purified B cells of RA patients and from healthy controls were incubated with fluorescein-loaded bifunctional NPs. Following the treatment with active or heat-inactivated human sera NPs, binding to live and dead B cells was detected by flow cytometry. The binding of fluorescein-loaded NPs was detected in FL1, while cells stained with the dead cell indicator To-Pro-3 were spotted in FL4. The small B-cell subset in RA samples showing double staining in the presence of active complement represents the β60-74Cit-specific B cells killed by the bifunctional NPs. This population was only detected in anti-Cit fibrin β-positive RA patients and was completely absent in healthy individuals (Fig. 5a). The number of dead cells was significantly lower in samples treated with heat-inactivated as compared with active sera, indicating the complement-dependent lysis of β60-74Cit-specific B cells (Fig. 5b).

## Discussion

We report here for the first time that the fibrin β60-74Cit peptide-specific B cells from RA patients can be specifically deleted in vitro by a bifunctional NP-based construct, where the β60-74Cit epitope and a HIV-1 gp120_233–247_ derivative peptide activating complement were covalently linked to NPs. Controlled clinical trials have shown that B-cell depletion therapies such as rituximab led to significant benefit for RA patients, indicating that B cells have a crucial role in the disease etiology [30–32, 34]. Although rituximab therapy only moderately affects pre-existing antibody titers, the patients are transiently immunosuppressed and have an increased risk of infection [34–36]. Therapies based on specific targeting and elimination of the autoantigen (e.g., citrullinated protein/peptide)-specific B cells would therefore be most advantageous.

Circulating plasma blasts/cells from RA patients secrete ACPA in vitro, indicating that in addition to the memory B-cell pool specific for citrullinated proteins/peptides there is an ongoing immune response in ACPA seropositive patients [39, 40]. Previous experiments revealed that the three Cit-containing fibrin β_60–74_ peptide represents the major ACPA epitope on the β chain of fibrin [11]. In our cohort 52 % of RA patients have shown serum antibody positivity with an average ELISA ratio around 10. Based on these data we selected patients with high antibody level for the cellular and functional assays.

Next, purified B cells from these selected patients were examined for β60-74Cit peptide positivity and for in vitro antibody production. Since the number of peptide-positive cells was expected to be low [40], and serum antibodies have low affinity to the citrullinated peptides [50, 51], the detection of peptide-specific cells is difficult. Therefore we stimulated B cells via TLR9, promoting proliferation independently from B-cell receptor and increasing the frequency of memory cells [49]. To increase the avidity of peptide binding to B cells and to improve detection of binding, we applied fluorescent microspheres coated with neutravidin and the biotinylated β60-74Cit peptide. Under these conditions we detected 2–3 % specific binding, indicating that β60-74Cit peptide-positive B cells are present in the circulation of RA patients and thus could be targeted by this peptide.

We also measured antibody secretion in the supernatants of ex vivo activated B cells, and in agreement with earlier results [39, 40] we found that even nonstimulated B cells from RA patients but not from healthy individuals secrete β60-74Cit peptide-specific antibodies that were significantly elevated due to stimulation with CpG. Interestingly, a low level of antibody production was observed with the control peptide (β60-74Arg) as well but that was not influenced by stimulation and was significantly lower in each sample as compared with the citrullinated peptide samples.

Depleting activity of antibody therapeutics is based on Fc-dependent effector functions such as antibody-dependent cytotoxicity and complement activating capacity inducing complement-dependent lysis [41, 42]. In order to combine the β60-74Cit peptide mimicking the autoantigen with an entity destroying the targeted B cells by complement-dependent lysis, we applied the HIV-1 gp120 derivative peptide CNNK. This peptide stimulated the classical pathway of complement activation and initiated the deposition of C3, C4, and C9 to the peptide-coated surfaces, at a degree comparable with IgG.

For targeted delivery of the effector, complement activating peptide and to increase the binding avidity of β60-74Cit peptide to B cells, we utilized biodegradable PLGA NPs that are not immunogenic, are nontoxic for cells, and are applicable under in vivo conditions [47]. NPs covered both with the effector peptide CNNQK and the targeting epitope peptide β60-74Cit were able to initiate the formation of soluble C5b-9 complex in human serum corresponding to the membrane attack complex, suggesting that the bifunctional NPs could induce cell lysis.

We postulated that these bifunctional NPs would indeed destroy B cells specific for the targeting compound, β60-74Cit peptide. Experiments testing the cytotoxic effect have shown that a small population (0.7–0.9 %) of B cells from RA patients having high serum antibody level bound NPs and a subset of these cells was killed in the presence of active complement.

Finally, as a proof of concept, the effect of bifunctional NPs was tested on ex vivo β60-74Cit peptide-specific antibody secretion. Depending on the serum concentration and the presence of active complement, the treatment of stimulated PBMCs with bifunctional NPs significantly reduced the number of β60-74Cit peptide-specific IgG-producing plasma cells in cultures obtained from RA patients. The number of plasma cells synthesizing nonspecific IgG in response to CpG stimuli was not modified significantly, demonstrating that the β60-74Cit epitope-specific autoreactive B cells are selectively depleted by the bifunctional NPs. Moreover, since this treatment diminishes citrullinated peptide-specific autoantibody production in vitro, it may result in a lower rate of autoreactive immune complex formation in seropositive patients, thus reducing inflammation. This pilot study only shows in vitro depletion of B cells recognizing a single epitope of fibrin, β60-74Cit. We suppose that a mixture of lytic NPs should be applied simultaneously, targeting several different autoantigen epitope-specific B cells. Since an autoantigen-specific depleting therapy affects only a small subset, it should leave the rest of the B cells unattended and enable them to respond to microbial stimuli.

## Conclusion

Taken together, the data of this pilot study demonstrate that biodegradable NPs armed with an epitope peptide (β60-74Cit) targeting B cells from RA patients and with an effector peptide (CNNQK) activating complement can destroy autoreactive, β60-74Cit-specific B cells in vitro. These data indicate that such constructs might be suitable for future development of personalized autoantigen-specific depletion therapy.

## Abbreviations

ACPA: Anti-citrullinated protein antibodies
ACR: American College of Rheumatology
ANOVA: Analysis of variance
BAFF: B-cell activating factor
Cit: Citrulline
ELISA: Enzyme-linked immunosorbent assay
ELISpot: Enzyme-linked immunospot assay
ESI-MS: Electrospray ionization mass spectrometry
EULAR: European League against Rheumatism
HIV-1: Human immunodeficiency virus-1
HPLC: High-performance liquid chromatography
HRP: Horseradish peroxidase
IL: Interleukin
MACS: Magnetic bead-activated cell sorting
NHS: Normal human serum
NP: Nanoparticle
OD: Optical density
PBMC: Peripheral blood mononuclear cell
PBS: Phosphate-buffered saline
PLGA: poly(d,l-lactic-*co*-glycolic acid)
RA: Rheumatoid arthritis
RF: Rheumatoid factor
SD: Standard deviation
TLR: Toll-like receptor

## References

[1] Gabriel SE (2001). The epidemiology of rheumatoid arthritis. Rheum Dis Clin North Am..

[2] Klareskog L, Ronnelid J, Lundberg K, Padyukov L, Alfredsson L (2008). Immunity to citrullinated proteins in rheumatoid arthritis. Annu Rev Immunol..

[3] Yamada R, Suzuki A, Chang X, Yamamoto K (2003). Peptidylarginine deiminase type 4: identification of a rheumatoid arthritis-susceptible gene. Trends Mol Med..

[4] Brink M, Hansson M, Mathsson L, Jakobsson PJ, Holmdahl R, Hallmans G (2013). Multiplex analyses of antibodies against citrullinated peptides in individuals prior to development of rheumatoid arthritis. Arthritis Rheum..

[5] Willemze A, Bohringer S, Knevel R, Levarht EW, Stoeken-Rijsbergen G, Houwing-Duistermaat JJ (2012). The ACPA recognition profile and subgrouping of ACPA-positive RA patients. Ann Rheum Dis..

[6] Perez ML, Gomara MJ, Ercilla G, Sanmarti R, Haro I (2007). Antibodies to citrullinated human fibrinogen synthetic peptides in diagnosing rheumatoid arthritis. J Med Chem..

[7] Nogueira L, Sebbag M, Vincent C, Arnaud M, Fournie B, Cantagrel A (2001). Performance of two ELISAs for antifilaggrin autoantibodies, using either affinity purified or deiminated recombinant human filaggrin, in the diagnosis of rheumatoid arthritis. Ann Rheum Dis..

[8] Vincent C, Nogueira L, Sebbag M, Chapuy-Regaud S, Arnaud M, Letourneur O (2002). Detection of antibodies to deiminated recombinant rat filaggrin by enzyme-linked immunosorbent assay: a highly effective test for the diagnosis of rheumatoid arthritis. Arthritis Rheum..

[9] Girbal-Neuhauser E, Durieux JJ, Arnaud M, Dalbon P, Sebbag M, Vincent C (1999). The epitopes targeted by the rheumatoid arthritis-associated antifilaggrin autoantibodies are posttranslationally generated on various sites of (pro)filaggrin by deimination of arginine residues. J Immunol..

[10] Sebbag M, Moinard N, Auger I, Clavel C, Arnaud J, Nogueira L (2006). Epitopes of human fibrin recognized by the rheumatoid arthritis-specific autoantibodies to citrullinated proteins. Eur J Immunol..

[11] Cornillet M, Sebbag M, Verrouil E, Magyar A, Babos F, Ruyssen-Witrand A (2014). The fibrin-derived citrullinated peptide beta60-74Cit(6)(0), (7)(2), (7)(4) bears the major ACPA epitope recognised by the rheumatoid arthritis-specific anticitrullinated fibrinogen autoantibodies and anti-CCP2 antibodies. Ann Rheum Dis..

[12] Iobagiu C, Magyar A, Nogueira L, Cornillet M, Sebbag M, Arnaud J (2011). The antigen specificity of the rheumatoid arthritis-associated ACPA directed to citrullinated fibrin is very closely restricted. J Autoimmun..

[13] Masson-Bessiere C, Sebbag M, Girbal-Neuhauser E, Nogueira L, Vincent C, Senshu T (2001). The major synovial targets of the rheumatoid arthritis-specific antifilaggrin autoantibodies are deiminated forms of the alpha- and beta-chains of fibrin. J Immunol..

[14] Bang H, Egerer K, Gauliard A, Luthke K, Rudolph PE, Fredenhagen G (2007). Mutation and citrullination modifies vimentin to a novel autoantigen for rheumatoid arthritis. Arthritis Rheum..

[15] Nicaise Roland P, Grootenboer Mignot S, Bruns A, Hurtado M, Palazzo E, Hayem G (2008). Antibodies to mutated citrullinated vimentin for diagnosing rheumatoid arthritis in anti-CCP-negative patients and for monitoring infliximab therapy. Arthritis Res Ther..

[16] Van Steendam K, Tilleman K, Deforce D (2011). The relevance of citrullinated vimentin in the production of antibodies against citrullinated proteins and the pathogenesis of rheumatoid arthritis. Rheumatology..

[17] Harre U, Georgess D, Bang H, Bozec A, Axmann R, Ossipova E (2012). Induction of osteoclastogenesis and bone loss by human autoantibodies against citrullinated vimentin. J Clin Invest..

[18] Kinloch A, Tatzer V, Wait R, Peston D, Lundberg K, Donatien P (2005). Identification of citrullinated alpha-enolase as a candidate autoantigen in rheumatoid arthritis. Arthritis Res Ther..

[19] Lundberg K, Kinloch A, Fisher BA, Wegner N, Wait R, Charles P (2008). Antibodies to citrullinated alpha-enolase peptide 1 are specific for rheumatoid arthritis and cross-react with bacterial enolase. Arthritis Rheum..

[20] Mahdi H, Fisher BA, Kallberg H, Plant D, Malmstrom V, Ronnelid J (2009). Specific interaction between genotype, smoking and autoimmunity to citrullinated alpha-enolase in the etiology of rheumatoid arthritis. Nat Genet..

[21] Burkhardt H, Sehnert B, Bockermann R, Engstrom A, Kalden JR, Holmdahl R (2005). Humoral immune response to citrullinated collagen type II determinants in early rheumatoid arthritis. Eur J Immunol..

[22] Suzuki A, Yamada R, Ohtake-Yamanaka M, Okazaki Y, Sawada T, Yamamoto K (2005). Anti-citrullinated collagen type I antibody is a target of autoimmunity in rheumatoid arthritis. Biochem Biophys Res Commun..

[23] Snir O, Widhe M, Hermansson M, von Spee C, Lindberg J, Hensen S (2010). Antibodies to several citrullinated antigens are enriched in the joints of rheumatoid arthritis patients. Arthritis Rheum..

[24] Haag S, Schneider N, Mason DE, Tuncel J, Andersson IE, Peters EC (2014). Identification of new citrulline-specific autoantibodies, which bind to human arthritic cartilage, by mass spectrometric analysis of citrullinated type II collagen. Arthritis Rheumatol..

[25] van Venrooij WJ, van Beers JJ, Pruijn GJ (2011). Anti-CCP antibodies: the past, the present and the future. Nat Rev Rheumatol..

[26] Anzilotti C, Merlini G, Pratesi F, Tommasi C, Chimenti D, Migliorini P (2006). Antibodies to viral citrullinated peptide in rheumatoid arthritis. J Rheumatol..

[27] Pratesi F, Tommasi C, Anzilotti C, Puxeddu I, Sardano E, Di Colo G (2011). Antibodies to a new viral citrullinated peptide, VCP2: fine specificity and correlation with anti-cyclic citrullinated peptide (CCP) and anti-VCP1 antibodies. Clin Exp Immunol..

[28] Laurent L, Anquetil F, Clavel C, Ndongo-Thiam N, Offer G, Miossec P (2015). IgM rheumatoid factor amplifies the inflammatory response of macrophages induced by the rheumatoid arthritis-specific immune complexes containing anticitrullinated protein antibodies. Ann Rheum Dis..

[29] Anquetil F, Clavel C, Offer G, Serre G, Sebbag M (2015). IgM and IgA rheumatoid factors purified from rheumatoid arthritis sera boost the Fc receptor- and complement-dependent effector functions of the disease-specific anti-citrullinated protein autoantibodies. J Immunol..

[30] Jacobi AM, Dorner T (2010). Current aspects of anti-CD20 therapy in rheumatoid arthritis. Curr Opin Pharmacol..

[31] Martinez-Gamboa L, Brezinschek HP, Burmester GR, Dorner T (2006). Immunopathologic role of B lymphocytes in rheumatoid arthritis: rationale of B cell-directed therapy. Autoimmun Rev..

[32] Sibilia J, Gottenberg JE, Mariette X (2008). Rituximab: a new therapeutic alternative in rheumatoid arthritis. Joint Bone Spine..

[33] Teng YK, Wheater G, Hogan VE, Stocks P, Levarht EW, Huizinga TW (2012). Induction of long-term B-cell depletion in refractory rheumatoid arthritis patients preferentially affects autoreactive more than protective humoral immunity. Arthritis Res Ther..

[34] Bingham CO, Looney RJ, Deodhar A, Halsey N, Greenwald M, Codding C (2010). Immunization responses in rheumatoid arthritis patients treated with rituximab: results from a controlled clinical trial. Arthritis Rheum..

[35] Anolik JH, Friedberg JW, Zheng B, Barnard J, Owen T, Cushing E (2007). B cell reconstitution after rituximab treatment of lymphoma recapitulates B cell ontogeny. Clin Immunol..

[36] Gottenberg JE, Ravaud P, Bardin T, Cacoub P, Cantagrel A, Combe B (2010). Risk factors for severe infections in patients with rheumatoid arthritis treated with rituximab in the autoimmunity and rituximab registry. Arthritis Rheum..

[37] Reparon-Schuijt CC, van Esch WJ, van Kooten C, Schellekens GA, de Jong BA, van Venrooij WJ (2001). Secretion of anti-citrulline-containing peptide antibody by B lymphocytes in rheumatoid arthritis. Arthritis Rheum..

[38] Szarka E, Babos F, Magyar A, Huber K, Szittner Z, Papp K (2014). Recognition of new citrulline-containing peptide epitopes by autoantibodies produced in vivo and in vitro by B cells of rheumatoid arthritis patients. Immunology..

[39] Bellatin MF, Han M, Fallena M, Fan L, Xia D, Olsen N (2012). Production of autoantibodies against citrullinated antigens/peptides by human B cells. J Immunol..

[40] Kerkman PF, Fabre E, van der Voort EI, Zaldumbide A, Rombouts Y, Rispens T, et al. Identification and characterisation of citrullinated antigen-specific B cells in peripheral blood of patients with rheumatoid arthritis. Ann Rheum Dis. 2015. 10.1136/annrheumdis-2014-207182 10.1136/annrheumdis-2014-207182 26034045

[41] Bologna L, Gotti E, Manganini M, Rambaldi A, Intermesoli T, Introna M (2011). Mechanism of action of type II, glycoengineered, anti-CD20 monoclonal antibody GA101 in B-chronic lymphocytic leukemia whole blood assays in comparison with rituximab and alemtuzumab. J Immunol..

[42] Melis JP, Strumane K, Ruuls SR, Beurskens FJ, Schuurman J, Parren PW. Complement in therapy and disease: regulating the complement system with antibody-based therapeutics. Mol Immunol. 2015;67(2 Pt A):117–30. 10.1016/j.molimm.2015.01.028 25697848

[43] Süsal C, Kirschfink M, Kropelin M, Daniel V, Opelz G (1996). Identification of complement activation sites in human immunodeficiency virus type-1 glycoprotein gp120. Blood..

[44] Aletaha D, Neogi T, Silman AJ, Funovits J, Felson DT (2010). Bingham 3rd CO, et al. Rheumatoid arthritis classification criteria: an American College of Rheumatology/European League Against Rheumatism collaborative initiative. Arthritis Rheum..

[45] Babos F, Szarka E, Nagy G, Majer Z, Sarmay G, Magyar A (2013). Role of N- or C-terminal biotinylation in autoantibody recognition of citrullin containing filaggrin epitope peptides in rheumatoid arthritis. Bioconjug Chem..

[46] Kiss E, Schnoller D, Pribranska K, Hill K, Penzes CB, Horvati K (2011). Nanoencapsulation of antitubercular drug isoniazid and its lipopeptide conjugate. J Disper Sci Technol..

[47] Horvati K, Bacsa B, Kiss E, Gyulai G, Fodor K, Balka G (2014). Nanoparticle encapsulated lipopeptide conjugate of antitubercular drug isoniazid: in vitro intracellular activity and in vivo efficacy in a Guinea pig model of tuberculosis. Bioconjug Chem..

[48] Abu-Rish EY, Amrani Y, Browning MJ (2013). Toll-like receptor 9 activation induces expression of membrane-bound B-cell activating factor (BAFF) on human B cells and leads to increased proliferation in response to both soluble and membrane-bound BAFF. Rheumatology..

[49] Jahnmatz M, Kesa G, Netterlid E, Buisman AM, Thorstensson R, Ahlborg N (2013). Optimization of a human IgG B-cell ELISpot assay for the analysis of vaccine-induced B-cell responses. J Immunol Methods..

[50] Ioan-Facsinay A, el-Bannoudi H, Scherer HU, van der Woude D, Menard HA, Lora M, et al. Anti-cyclic citrullinated peptide antibodies are a collection of anti-citrullinated protein antibodies and contain overlapping and non-overlapping reactivities. Ann Rheum Dis. 2011;70:188–93. 10.1136/ard.2010.131102 20736390

[51] Ossipova E, Cerqueira CF, Reed E, Kharlamova N, Israelsson L, Holmdahl R (2014). Affinity purified anti-citrullinated protein/peptide antibodies target antigens expressed in the rheumatoid joint. Arthritis Res Ther..

